# Visceral Pain: Basic Research Concepts and Therapeutic Interventions

**DOI:** 10.3844/ijrnsp.2015.27.28

**Published:** 2015

**Authors:** Victor V. Chaban

**Affiliations:** Charles R. Drew University of Medicine and Science, University of California, Los Angeles, USA

The accumulation of nociceptive diseases that limit normal body functions is a major risk factor for a disability, and visceral pain is one of the most prevalent human health problems. Additionally, many pain-associated diseases are accompanied by the concomitant decline in cognitive and motor performance. The complex interplay and balance between diverse signal transduction mediators, genetic background and environmental factors may ultimately determine the outcome of nociceptive progression in various disorders. Pain is a subjective feeling that is difficult to standardize and parameterize in a traditional fashion for scientific analysis. The causes of visceral pain are often not very clear, as there are many symptoms of the reproductive, gastrointestinal, musculoskeletal, neurological, psychological systems and urinary tract that often cooccur in the same patient. VIscero-somatic and visceroviseral hyperalgesia and allodynia Cause the perception of pain to spread for an initial area, to adjacent visceral sites (Pan and Malykhina, 2014). Often times, there is not a clear relationship between the severity. There is often no clear relationship between the severity of the visceral pain and pathology in the viscera, including the reproductive tract, urinary bladder and colon. The clinician treating this pain is often times tempted to adopt a unidimensional approach, focusing on one organ system, ignoring the psychological and behavioral manifestations of the visceral pain. Therefore, studies of the nervous system in individuals with visceral pain associated with reproduction such as Chronic Pelvic Pain (CPP) syndrome, urinary system such as Painful Bladder Syndrome (PBS) and bowel disorders such as Irritable Bowel Syndrome (IBS), suggest a model in which alteration in the central stress circuits in predisposed individuals may trigger and then maintain, the pain and pathophysiological changes in the viscera (Mayer, 2011). These patients have significantly more depression, psychological and somatic complains and more often give a history of physical, sexual or emotional abuse, or trauma. Chronic visceral pain results in adverse affects not only one’s mood, but also their professional and social lives, as well as general well-being;; the quality of life issues can affect the severity of pain, degree of impairment resulting from a painful condition and success of treatment modalities in alleviating pain.

Pain accounts for a majority of all primary health care visits. For the past decade, medical literature has carefully documented the under-treatment of all types of pain by physicians. Pain is a complex and individual experience that is often difficult for patients to fully describe using a conventional clinical assessment (Meltzak, 2001). Visceral pain affects up to 25% of women at some time in their lives (about a billion worldwide) and can result in dysmenorrhea, dyspareunia, menstrual irregularities, back pain, gastrointestinal and genitourinary symptoms and reduced fecundity. The incidents of persistent visceral pain associated with functional disorders such as IBS, CPP, PBS and others is 2–3 times higher in women than in men, suggesting estrogen modulation. In women, pain symptoms and nociceptive thresholds vary with reproductive cycle and our previous data strongly suggest the role of estrogen receptors in modulating of nociceptive signaling (Chaban *et al*., 2011; Cho and Chaban, 2012; Chaban, 2012; 2014).

There are two essential components of pain: discriminative and affective. The discriminative component includes the ability to identify the stimulus as originating from somatic or visceral tissue, determine some of the physical properties of the stimulus and localize it in space, time and along a continuum of intensities. The affective component is the experience which motivates escape, avoidance and protective behavior. All of these components of pain must be considered in any discussion of the neurophysiological basis of visceral pain. Because of the inherent subjectivity of pain, there is a wide disparity among individuals in the way that they experience pain generated by what seem to be similar stimuli. There is also a tension between the subjectivity of the patient’s pain experience and the common insistence of the clinician upon objective findings that are proportionate with the patient’s complaints, to enable to distinguish between exaggerated pain reports. Proposed therapeutic considerations must also include the neural systems modulating pain, for it is well known that pain can be profoundly influenced by other somatic stimuli and by attentional, emotional and cognitive factors. Careful history and physical examination are crucial in evaluating a suffering patient and must address all of the possible systems potentially involved in visceral pain.

An important focus of clinical management now includes the assessment of pain on various aspects of a patient’s existence. The health-related quality of life that encompasses Health related qualities of life are comprised of aspects of health and well-being that are valued by patients, such as their emotional, physical, and cognitive state, and ability to participate in meaningful tasks. There is a concern that not enough emphasis is placed on a clinical validity (i.e. issues which are important to patients and reflect their experiences). A balance between biomedical, organ-oriented and cognitive interpersonal approaches is the most appropriate to study this psychosomatic interface. In view of the iatrogenic component in the maintenance of painful syndromes, clinician-centered interventions and close observation of the clinician-patient relationship are of particular importance. Nociceptive responses involve a vast number of messenger molecules that interact with enzymes and receptors of all classes. They direct the recruitment of different types of cells to assist in the recovery of a health state. A balance between these messengers and the redundancy of various body systems presents major difficulties for therapeutic intervention. Nevertheless, it is a very important aspect to consider in the treatment of disorders association with visceral pain.

## References

[R1] Pan XQ, Malykhina AP (2014). Estrous cycle dependent fluctuations of regulatory neuropeptides in the lower urinary tract of female rats upon colon-bladder cross-sensitization. PLOS One.

[R2] Mayer E (2011). Gut feelings: The emerging biology of gut-brain communication. Nat. Rev. Neurosci.

[R3] Meltzak R (2001). Pain and the Neuromatrix in the Brain. J. Dent. Educ.

[R4] Chaban V (2012). Estrogen and visceral nociception at the level of primary sensory neurons. Pain Res. Treat.

[R5] Chaban V, Li J, McDonald JS, Rapkin A, Micevych P (2011). Estradiol attenuates the adenosine triphosphate-induced increase of intracellular calcium through group II metabotropic glutamate receptors in rat dorsal root ganglion neurons. J. Neurosci. Res.

[R6] Cho T, Chaban VV (2012). Interaction Between P2X3 and Oestrogen Receptor (ER)F/ERG in ATP-mediated calcium signalling in mice sensory neurones. J. Neuroendocrinol.

[R7] Chaban V (2014). Chronic pelvic pain: Focus on etiology and modulation. Int. J. Res. Nursing.

